# Metatranscriptomic Analysis and In Silico Approach Identified Mycoviruses in the Arbuscular Mycorrhizal Fungus *Rhizophagus* spp.

**DOI:** 10.3390/v10120707

**Published:** 2018-12-12

**Authors:** Achal Neupane, Chenchen Feng, Jiuhuan Feng, Arjun Kafle, Heike Bücking, Shin-Yi Lee Marzano

**Affiliations:** 1 Department of Biology and Microbiology, South Dakota State University, Brookings, SD 57007, USA; achal.neupane@sdstate.edu (A.N.); jiuhuan.feng@sdstate.edu (J.F.); arjun.kafle@sdstate.edu (A.K.); heike.bucking@sdstate.edu (H.B.); 2 Department of Agronomy, Horticulture, and Plant Sciences, South Dakota State University, Brookings, SD 57007, USA; chenchen.feng@sdstate.edu

**Keywords:** mycorrhizal fungi, mycovirus, mitovirus, *Rhizophagus*

## Abstract

Arbuscular mycorrhizal fungi (AMF), including *Rhizophagus* spp., can play important roles in nutrient cycling of the rhizosphere. However, the effect of virus infection on AMF’s role in nutrient cycling cannot be determined without first knowing the diversity of the mycoviruses in AMF. Therefore, in this study, we sequenced the *R*. *irregularis* isolate-09 due to its previously demonstrated high efficiency in increasing the N/P uptake of the plant. We identified one novel mitovirus contig of 3685 bp, further confirmed by reverse transcription-PCR. Also, publicly available *Rhizophagus* spp. RNA-Seq data were analyzed to recover five partial virus sequences from family *Narnaviridae*, among which four were from *R. diaphanum* MUCL-43196 and one was from *R. irregularis* strain-C2 that was similar to members of the *Mitovirus* genus. These contigs coded genomes larger than the regular mitoviruses infecting pathogenic fungi and can be translated by either a mitochondrial translation code or a cytoplasmic translation code, which was also reported in previously found mitoviruses infecting mycorrhizae. The five newly identified virus sequences are comprised of functionally conserved RdRp motifs and formed two separate subclades with mitoviruses infecting *Gigaspora* *margarita* and *Rhizophagus* *clarus*, further supporting virus-host co-evolution theory. This study expands our understanding of virus diversity. Even though AMF is notably hard to investigate due to its biotrophic nature, this study demonstrates the utility of whole root metatranscriptome.

## 1. Introduction

About eighty percent of land plants form symbiotic relationships with arbuscular mycorrhizal (AM) fungi [1], where obligate mutualistic fungi colonize plant roots for their spores to germinate and form hyphae. Examples of endophytic fungi, including AM fungi, have been shown to help control fungal pathogens [2], resist drought and salinity [3,4], and affect the overall fitness (growth, survival, etc.) of vascular plant families [5,6]. However, it is not well known whether multipartite plant-AM fungi-virus interactions may play a role in the plant’s adaptation to biotic and abiotic stresses. Specifically, it remains unclear how AM fungi infections can alter patterns of plant gene expression, or whether superimposed viral infections would have cascading effects on the plant gene expression.

As AM fungi play important roles in carbon/nitrogen/phosphate cycling and compete with pathogens for ecological niches, there is emerging interest in discovering whether they harbor viruses through next generation sequencing [7,8]. It is necessary to recover the virus sequences associated with these fungi before further determining the effect of viral infections on hyphal growth and nutrient uptake of the host plant. Other endophytic fungi forming mutualistic symbiotic relationships with land plants have been shown to harbor viruses and confer heat tolerance when infected by virus(es) [9]. However, the prevalence and effects of virus infection on AM fungi are largely unknown, and the roles they play in the context of carbon/nutrient cycling are still ambiguous. Additionally, the virome of AM fungi is difficult to study partly because of its obligate nature of biotrophic reproduction that requires a large number of hyphae [8] or spores [10].

Likely not mutually exclusive, “virus-host ancient coevolution theory” is one of two hypotheses that have been proposed for mycovirus origin [11], with the other hypothesis suggesting that plant viruses are the origin of mycoviruses [12]. The *Narnaviridae* family of mycovirus is comprised of two genera, namely *Narnavirus* and *Mitovirus*, and include some of the simplest RNA viruses ever identified [13]. Narnaviruses are known to be localized in the cytosol, expressed using standard genetic code [13] and likely evolved from a RNA bacteriophage [14]. Mitoviruses, meanwhile, are known to be found primarily in mitochondria of the fungal host, translated using mold mitochondrial genetic code, and are believed to have evolved as endosymbiont of alphaproteobacteria [13]. Additionally, *Narnaviradae* RdRps are closely related to leviviruses, viruses of bacteria and ourmiaviruses of plants [13,14,15].

Typical mitoviruses have <3 kb genomes and have been detected in both fungi and plants [16], and either exist endogenously in plant genomes or freely replicate in mitochondria as genuine viruses. Endogenous mitovirus sequences may or may not be transcribed actively [17]. However, mitoviruses detected from mycorrhizal fungi generally have genome sizes greater than 3 kb, and the coding regions can be either translated by a cytosolic/nuclear genetic codon usage table or a mitochondrial table [7,18].

We recently screened soybean leaf-associated viromes and identified 23 nearly full-length mycoviral genomes using RNA-Seq of total RNA even when the plant sequences were present [19]. In order to understand the effects of a tritrophic relationship among plant-AM fungi-virus interactions on soil processes, root-associated viromes should be profiled. Differences in phosphate and nitrogen uptake of AMF were observed even within the same species [20], suggesting that besides genetic variability, there could be microbes, including mycoviruses, hosted by AMF that affect their functions. Notably, Ikeda et al. [21] determined that AM fungi infected by the virus, GRF1V-M, produced two-fold fewer spores compared to the virus-free culture line of *Rhizophagus* spp. strain RF1, and was less efficient in promoting plant growth. Therefore, in this study, we aimed to discover and characterize new mycoviruses infecting AM fungi with combined approaches. We used a culture-independent metatranscriptomics approach to detect viruses infecting *Rhizophagus* spp., and by reanalyzing data from other *Rhizophagus* spp. available as SRR data from the NCBI database (https://www.ncbi.nlm.nih.gov/sra). As *Medicago truncatula* is a host plant for *Rhizophagus* spp., we performed metatranscriptome RNA-Seq on *M*. *truncatula* roots directly to screen for mycoviruses. This research could provide insight on virus evolution and may help researchers form hypotheses to study the mechanisms of the varying functions from isolates/species of AMF that affect their biofertilizer potential. 

## 2. Materials and Methods

### 2.1. Plant and Fungal Material

*Medicago truncatula* (A17) seeds were surface sterilized with concentrated H_2_SO_4_, rinsed with autoclaved distilled water, and kept at 4 °C overnight. The seeds were then pregerminated on moist filter paper for 7 days until fully grown cotyledons were developed. We transferred the seedlings into pots containing 250 mL of an autoclaved soil substrate mixture of 40% sand, 20% perlite, 20% vermiculite, and 20% soil (v:v:v:v; 4.81 mg/kg P_i_ after Olsen extraction, 10 mg/kg NH_4_^+^, 34.40 mg/kg NO_3_^−^, pH 8.26). At transplanting, each seedling was inoculated with 0.4 g mycorrhizal root material and ~500 spores of *Rhizophagus irregularis* N.C. Schenck & G.S. Sm. (isolate 09 collected from Southwest Spain by Mycovitro S.L. Biotechnología ecológica, Granada, Spain). The roots and the fungal inoculum were produced in axenic Ri T-DNA transformed carrot (*Daucus* *carota* clone DCI) root organ cultures in Petri dishes filled with mineral medium [22]. After approximately eight weeks, the spores were isolated by blending the medium in 10 mM citrate buffer (pH 6.0).

The plants were grown in a growth chamber with a 25 °C/20 °C day and night cycle, 30% humidity, a photosynthetic active radiation of 225 μmol m^−2^ s^−1^, and watered when necessary. After seven weeks, the plants were harvested, and mycorrhizal root material was frozen in liquid nitrogen and stored at −80 °C until RNA extraction. To quantify the mycorrhizal colonization, some roots were cleared with 10% KOH solution at 80 °C for 30 min, rinsed with water, and stained with 5% ink at 80 °C for 15 min [23]. We analyzed a minimum of 100 root segments to determine the percentage of AM root colonization by using the gridline intersection method [24].

### 2.2. High-Throughput Sequencing

Approximately 150 mg of root tissue was ground in liquid nitrogen, and total RNA was extracted using the Qiagen RNeasy Plant Mini Kit (Valencia, CA, USA). RNA samples were treated with DNase I, evaluated for integrity by agarose gel electrophoresis, and rRNAs were removed by the Ribo-Zero Plant Kit (Illumina, San Diego, CA, USA), and used as templates to construct the library with a ScriptSeq RNA sample preparation kit (Illumina, San Diego, CA, USA). The library was submitted to the W. M. Keck Center, University of Illinois for quality check and cleanup and sequenced on an Illumina HiSeq 4000 for 100 bp paired-end reads.

### 2.3. Sequence Analysis

Sequence reads from the above sequencing run, as well as publicly available data (published by Tisserant et al., 2013 [25]) under SRX312982 (*Rhizophagus diaphanum* MUCL 43196; previously *Glomus diaphanum* [26]), SRX375378 (*Rhizophagus irregularis* DAOM-197198; previously *Glomus intraradices* or *Rhizophagus intraradices* [25,26]) and SRX312214 (*Rhizophagus irregularis* C2) were retrieved from the NCBI database and the paired-end sequence reads (100 nt in length) were trimmed by BBMap tools (https://sourceforge.net/projects/bbmap) and assembled into contigs using the TRINITY de novo transcriptome assembler [27]. Contigs with significant similarity to viral amino acid sequences were identified using USEARCH ublast option [28] with a parameter e-value of 0.0001 and compared to a custom database containing *Rhizophagus irregularis* and viral amino acid sequences from GenBank using BLASTX [29]. The nucleotide sequences of all suspected mycovirus contigs were compared with the NCBI nr database using BLASTX [29] to exclude misidentified sequences. The number of reads aligning to different target sequences was calculated using Bowtie [30]. Predicted amino acid sequences were aligned using ClustalW [31]. Aligned protein sequences were used to reconstruct a maximum likelihood tree with the model WAG + G + I + F using Mega (Molecular Evolutionary Genetics Analysis) version 7.0 software [32]. Statistical support for this analysis was computed based on 100 nonparametric bootstrap replicates. The MEME suite 5.0.1 was used to compare the motifs [33]. The viral sequences were submitted to the GenBank database under the following accession numbers: RdMV1, MH732931; RdMV2, MH732930; RdMV3, MK156099; RdMV4, MK156100; RirMV1 and MH732933.

### 2.4. Reverse-Transcription PCR (RT-PCR)

To confirm that the RirMV1 sequence detected was not an artifact and indeed derived from the *Medicago* root material, RT-PCR amplified a 3.4 kb amplicon from the RNA extract after DNase treatment by the virus-specific primers, RirMV1-197F (5′-CACCTATGAGCCCGGTTAAA-3′) and RirMV1-3409R (5′-GGAGAATCGTCCTTCCTTCC-3′). For the nested PCR the primers RirMV1-197F and RirMV1-3228R (5′-ACCTTTCCAGGGGAGACCTA-3′) were used. The nested amplicon was submitted for Sanger sequencing to confirm the identity after ExoSap-IT cleanup (Thermofisher, Waltham, MA, USA). Additionally, to confirm that the viral sequence is not from the Medicago host, reverse transcription of cDNA was made by using Maxima H Minus Reverse Transcriptase (Thermo Scientific, Waltham, MA, USA) at 50 °C for 30 min followed by 85 °C for 5 min inactivation. Then PCR was performed using RirMV1-197F and RirMV1-3228R primer set and Phire Plant Direct PCR Kit (Thermo Scientific, Waltham, MA, USA).

### 2.5. Rapid Amplification of cDNA Ends (RACE)

To complete the genome sequence of RirMV1, the 5′- and 3′-terminal sequences were determined using the FirstChoice RLM-RACE (rapid amplification of cDNA ends) kit (Life Technologies). Primers 336R (5′-AGAGCGGTCGCTTCTGTCTA-3′) and 216R (5′-TTTAACCGGGCTCATAGGTG-3′) were used for 5′-RACE as outer and inner primers, respectively. Primers 3210F (5′-TAGGTCTCCCCTGGAAAGGT-3′) and 3347F (5′-CGACCTCTGGAGGTTGAAAG-3′) were used for 3′-RACE as outer and inner primers, respectively.

## 3. Results

### 3.1. Mycoviruses in the Metatranscriptome of Rhizophagus Irregularis Inoculated Roots

**Figure 1:**
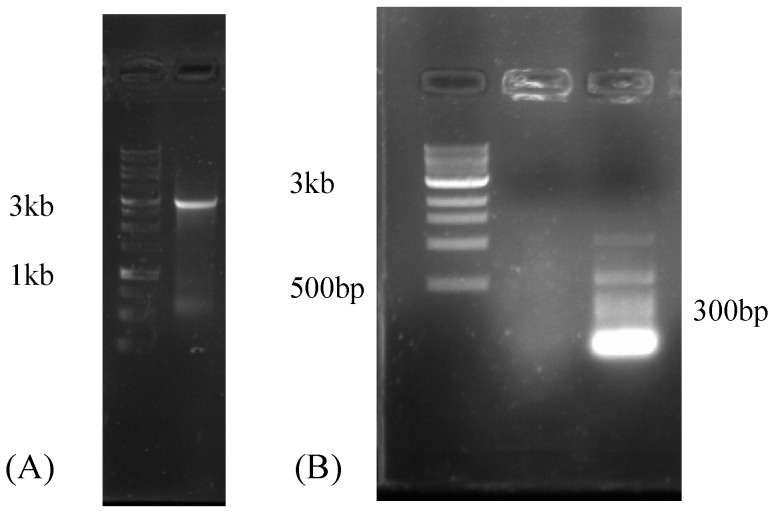
(**A**) Agarose gel electrophoresis of the RT-PCR product showing a ~3 kb nested PCR amplicon that was confirmed by Sanger sequencing as cDNA amplicon of RirMV1. Left lane: 1 kb ladder. Right lane: RirMV1 amplicon of the predicted size of 3 kb and (**B**) Agarose gel electrophoresis of the RT-PCR product showing no amplification, suggesting the viral contig of RNA-Seq was not originated from *Medicago* root without *R*. *irregularis* strain 09 infection. Left to right lanes: 1 kb ladder, viral primers, plant primers.

After colonization of *R*. *irregularis* was confirmed by microscopy (Figure S1), sequencing of the mycorrhizal *Medicago truncatula* roots on the Illumina HiSeq4000 platform resulted in a total of 85 million paired-end reads, yielding 12.1 GB of sequence information. The data were submitted to the SRA database at NCBI (accession number SRX4679168). In this data set, we identified one viral contig (RirMV1). To confirm the viral contig assembled from the short reads, RirMV1-3409R primed cDNA was used as a PCR template to amplify most of the viral contig. The primers RirMV1-197F and RirMV1-3409R amplified multiple bands, and among them there was a faint 3 kb band (not shown). The 3 kb band was subsequently excised, and the gel was purified. Nested PCR using RirMV1-197F and RirMV1-3228R resulted in a clear band of 3 kb (Figure 1A). Sanger sequencing using the same primer set confirmed the band as RirMV1 cDNA amplicon. PCR attempts to amplify RirMV1 directly from the DNA of the *R*. *irregularis* strain 09 infected *Medicago* roots were not successful, indicating that the viral transcript was not derived from virus segments integrated into the host genome that are actively expressed. Instead they are from the genuine virus (Figure 1B). We also attempted to amplify a smaller target using viral-specific primers 197F and 336R for 140 bp amplicon and ran a 1.5% gel to confirm that there was no amplification, leading to the same conclusion that the viral sequence was not from *Medicago*, which confirms that *R*. *irregularis* is the host of the virus. Additionally, we also attempted RACE amplification of RirMV1 contig, but failed to extend the contig.

### 3.2. Mycoviruses in the Transcriptomes of Rhizophagus spp.

To identify mycoviruses in *Rhizophagus* spp., we first reanalyzed the publicly available RNA-Seq data sets of *R*. *irregularis* strain-C2 (SRX312214), *R*. *irregularis* DAOM-197198 (SRX375378) and *R. diaphanum* MUCL 43196 (SRX312982). No viruses could be identified in the available *Rhizophagus irregularis* DAOM-197198 transcriptome, but we found multiple novel mycoviruses in the transcriptome of *R*. *diaphanum* MUCL 43196 (Rhizophagus diaphanum mitovirus 1—RdMV1, 3554 nt long; Rhizophagus diaphanum mitovirus 2—RdMV2, 4382 nt long; Rhizophagus diaphanum mitovirus 3—RdMV3, 3652 nt long and Rhizophagus diaphanum mitovirus 4—RdMV4, 3443 nt long) that had similarities to members of the *Mitovirus* genus (Table 1).

Overall, RirMV1 had 41,322 reads and a 0.10% alignment with the sequencing run of the colonized *Medicago* roots. Among the total reads for SRX312982 from *R*. *diaphanum*, 1475 read-counts aligned to RdMV1 (0.0015%), 3,649 to RdMV2 (0.0036%), 2350 to RdMV3 (0.0023%), and 462 to RdMV4 (0.00045%), see Table 1. The NCBI BLAST results indicate that these contigs are putatively similar in function to previously identified RNA-dependent RNA polymerases of mitoviruses.

### 3.3. Phylogenetic Analysis and the Characterization of Conserved RdRp Region of Mitoviruses

**Figure 2:**
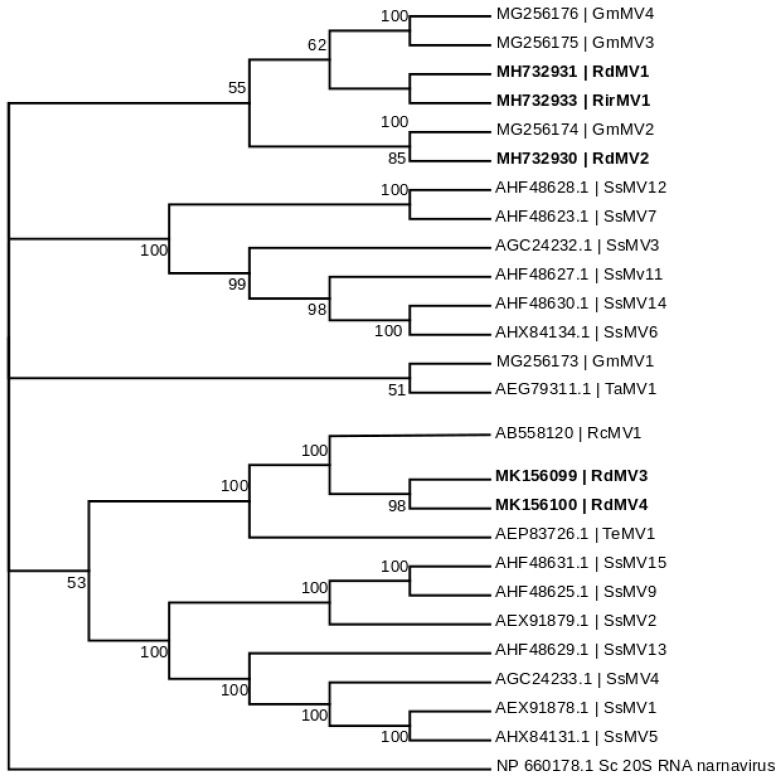
Maximum likelihood tree (with bootstrap consensus) depicting the relationships of the predicted amino acid sequences of RNA-dependent RNA polymerase (RdRp) of the *Rhizophagus* mitoviruses, and other confirmed and proposed members of the *Narnaviridae*. Predicted RdRp amino acid sequences were aligned with ClustalW [31], and the phylogenetic tree was inferred using Mega 7.0 software [32]. Branch lengths are scaled to the expected underlying number of amino acid substitutions per site. The Saccharomyces 20S RNA narnavirus RdRp amino acid sequence was used as an outgroup to root the tree. Five newly identified mitoviruses (in bold) formed two separate monophyletic clusters between the Rhizophagus-associated mitoviruses. The following abbreviations were used for the Mitovirus (MV) sequences: Sc, *Saccharomyces cerevisiae*; Gm, *Gigaspora margarita*; Rd, *Rhizophagus diaphanum*; Rc, *Rhizophagus clarus*; Sc, *Sclerotinia sclerotiorum*; Rir, *Rhizophagus irregularis;* Ta, *Tuber aestivum*; Te, *Tuber excavatum*.

**Figure 3:**
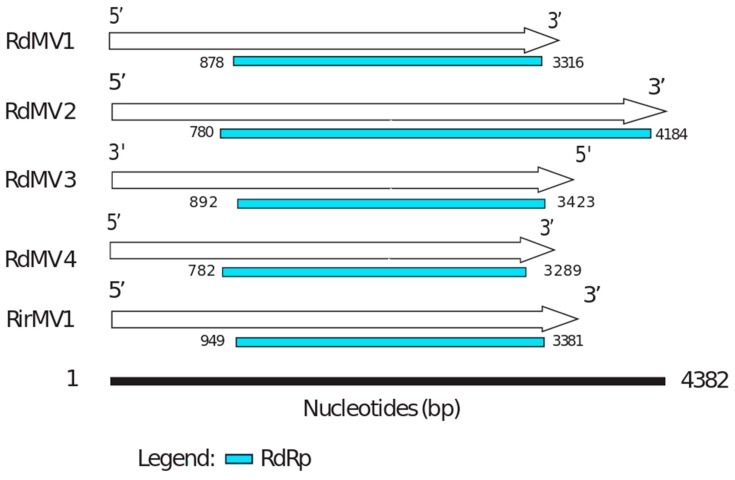
The genome organization of *Rhizophagus* spp. mitoviruses. The comparisons are of the organizations of RdMV1, RdMV2, RdMV3, RdMV4 and RirMV1. RdRp coding regions are labeled in blue (see also Table 1).

**Figure 4:**
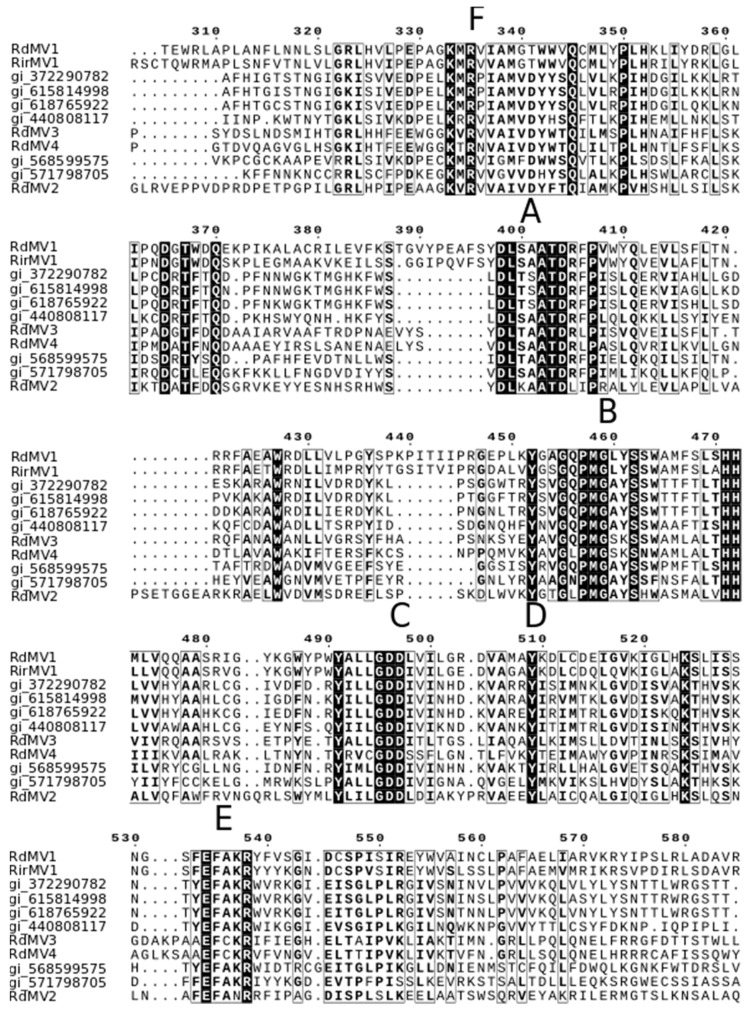
Conserved motifs identified in the RdRp domain of the genus *Mitovirus* based on the multiple sequence alignment of the amino acid sequences. Similar to the other mitoviruses, six conserved motifs were found. These conserved regions were labeled A-F as RdRp associated motifs described previously [36].

To identify the evolutionary lineages among the identified mitoviruses, we analyzed the protein sequences of mitoviruses to reconstruct the phylogenetic tree (Figure 2). While there was no virus found in SRX375378 and SRX312214, there were four partial genome sequences identified from SRX312982 publicly available data similar to viruses from the family *Narnaviridae.* Two of these sequences (RdMV1 and RdMV2) formed a separate clade with RirMV1 and previously identified mitoviruses from *Gigaspora margarita* (GmMV2, GmMV3, and GmMV4). The other two contigs (RdMV3 and RdMV4) were phylogenetically similar to the mitovirus infecting *Rhizophagus clarus* (RcMV1) (Figure 2). We also compared the genome structure of identified mitoviruses to see if the RdRp region is uniformly conserved (Figure 3). To confirm the presence of functionally conserved motifs of RNA-dependent RNA polymerase (RdRp) in identified viruses, we further analyzed and compared six RdRp motifs (A–F) with other mitoviruses of pathogenic fungi in the NCBI database (Figure 4). Three of these motifs (A–C) are among the most conserved motifs of RdRp and include residues involved in catalytic activation and dNTP/rNTP recognition (discussed in detail below) by RdRp [34,35]. Noticeable differences in the amino acid sequence include a histidine in the mitoviruses of *Rhizophagus* spp. instead of a serine at residue 325 and a glutamic acid instead of an aspartic acid at residue 329 within the RdRp motif F.

The RdRps can be translated using either a cytosolic or mitochondrial code. The complete coding RdRp was 811 aa long for RirMV1, compared to the average of ~700 aa for the most closely related mitoviruses infecting *Sclerotinia sclerotiorum*. RdMV1 and RirMV1 have nearly identical lengths of RdRp, these being 812 aa and 811 aa, respectively.

## 4. Discussion

Our own studies revealed a high intraspecfic diversity in the growth and nutrient uptake benefits after colonization with different AM isolates [37]. As we report the identification of mycoviruses in a lab culture and *in silico* from *Rhizophagus* spp., it would be interesting to determine whether mitoviruses play any role in the variability of these responses. In this study, we identified a novel mitovirus from the sequenced transcriptome of *R*. *irregularis* and confirmed the presence of RT-PCR amplicon of 3kb with gel electrophoresis (Figure 1A). Additionally, agarose gel electrophoresis of the RT-PCR product from the non-mycorrhizal *Medicago* root without *Rhizophagus* infection showed no amplification of viral contig, suggesting the newly identified viral contig (RirMV1) was not from the plant, but from *R*. *irregularis* (Figure 1B). We also identified four novel mitoviruses: RdMV1, RdMV2, RdMV3 and RdMV4 from *R*. *diaphanum* from the publicly available SRA database in NCBI (Table 1).

After the viral contigs were assembled, RT-PCR was used to verify the presence of any putative viral sequence. Also, it is necessary to rule out the possibility that the putative mycovirus genomes identified in this study could have been derived from mycoviruses integrated into the AMF genome. Oligonucleotide primers specific to the putative viral sequences need to be used to amplify the sequences using fungal genomic DNA as the template. The infection of these viruses could have resulted in beneficial/neutral effects on the host as they were selected to be sequenced without regards to apparent abnormal growth. These novel viral sequences may be used to establish Koch’s postulates in future studies and to provide bases for mechanisms responsible in different nutrient uptake and plant biomass responses.

Using a mitochondrial translation table, the amino acid sequences of the three of five predicted mitovirus-like contigs clustered with mitoviruses of filamentous fungi, and constituted two distinct subclades along with mitoviruses infecting *G*. *margarita* (Figure 2). Our analysis showed that RdMV3 and RdMV4 are closely related to Rhizophagus clarus mitovirus 1-RF1 (RcMV1; AB558120) which is closely related to another mitovirus that was found in the ectomycorrhizal fungus *Tuber excavatum* (TeMV1; AEP83726.1). Their corresponding fungal hosts are all AM fungi and these mitoviruses all have distinctly longer RdRps than other mitoviruses that use mitochondrial translation code only. Similar to what was reported by Ikeda et al. [21], we found that the largest ORF of the two mitoviruses can also be predicted by applying the universal genetic code. Generally, functional translation of RdRp in mitoviruses involves activation of a mitochondrial genetic code [38], and as a result, tryptophan residues in mitoviruses (such as TeMV, CpMV, and HmMV1-18) are coded either by a UGA (which in universal genetic code means termination) or a UGG codon [7]. Recently identified mitoviruses from AMF, including RcMV1-RF1 [7] and the mitoviruses identified in *G. margarita* [10], all use the UGG codon for tryptophan which is compatible with both cytoplasmic and mitochondrial translation. All five novel mitoviruses identified from our study were found to use UGG for tryptophan. Interestingly, Nibert (2017) provided sequence-based explanation of this subgroup of mitoviruses using UGG instead of UGA for tryptophan in mitochondria, which is likely due to the mitochondrial codon of UGA for tryptophan in the respective fungal hosts that is correspondingly rare. Therefore, Nibert (2017) speculated that this unique group of mitoviruses do not actually replicate in cytosol [39].

Our results support the virus-host coevolution theory for the origin of these mitoviruses infecting nonpathogenic AMF fungi because the viruses they harbor do not cluster with mitoviruses from pathogenic fungi. First, amino acid sizes for the RdRps are very similar between the three viruses, RirMV1, RdMV1 and RdMV3, and the RdRp motifs are highly conserved between them (Figure 3). Besides the conserved motifs I to IV identified in Kitahara et al. [7] and Gorbalenya et al. [34], we also identified the motifs D and E (Figure 4) as shown in Bartholomaus et al. (2016) [36]. Out of these six motifs, A, B and C are among the most conserved structural motifs of the palm subdomain of RdRp with active catalytic sites [34]. Motif A (DX_5_D) contains two aspartic acid residues separated by any five amino acids, while motif C contains two aspartic acid residues, consecutively. These residues are known to form divalent bonds between Mg^2+^ ions and/or Mn^2+^ ions for catalytic activation of the domain [34]. Similarly, motif B is known to form a long and conserved alpha-helix sequence with an asparagine residue which is indispensable for discriminating dNTPs and rNTPs that determine whether DNA or RNA is produced [35]. Although these motifs are known to be required for polymerase activity, the other three motifs (D, E and F) are not well studied in terms of their function.

Experiments to assess the impacts of viral infection on fungal colonization and sporulation and on the ability of virus-infected fungal isolates to affect nutrient cycling in host crops can be done by using virus-induced gene silencing (VIGS) approaches to knockdown the expression of viral transcripts. VIGS systems have been successfully applied in *R*. *clarus* to study fungal gene functions [40], which could be modified to instead silence RirMV1 using *Nicotiana benthamiana* as the plant host to deliver the silencing construct through the Cucumber mosaic virus Y strain based VIGS system [41]. The silencing effect will be effective if RirMV1 replicates in the cytoplasm as well, but will be ineffective if it replicates only in mitochondria since the RNA silencing machinery is not present in mitochondria and the double-layered membrane is a barrier. This may also resolve the long-standing question of whether mitoviruses in AMF replicate in the cytoplasm, which can shed light on the evolution of capsidless positive-strand RNA viruses.

## Figures and Tables

**Table 1:** Identified mycovirus-like sequences, contig lengths, and their putative functions are shown in the table below, including the data source from which the virus sequence was recovered. These new contigs were identified as mitoviruses (MV) and were recovered from two different fungal hosts (Rd, *Rhizophagus diaphanum*; Rir, *Rhizophagus irregularis*).

Contig Name	Data Source	Contig Length (nt)	Read Counts	NCBI Accession	Amino Acid Identity (%)	Putative Function (Most Similar Virus)
RdMV1	SRX312982 (*Rhizophagus diaphanum* MUCL 43196)	3554	1475	MH732931	32	RNA-dependent RNA polymerase [Rhizoctonia solani mitovirus 12]
RdMV2	SRX312982 (*Rhizophagus diaphanum* MUCL 43196)	4382	3649	MH732930	28	RNA-dependent RNA polymerase [Gigaspora margarita mitovirus 2]
RdMV3	SRX312982 (*Rhizophagus diaphanum* MUCL 43196)	3652	2350	MK156099	36	RNA-directed RNA polymerase [Rhizophagus sp. RF1 mitovirus]
RdMV4	SRX312982 (*Rhizophagus diaphanum* MUCL 43196)	3443	462	MK156100	30	RNA-dependent RNA polymerase [Rhizoctonia mitovirus 1]
RirMV1	Sequenced transcriptome (submitted under accession: SRX4679168)	3685	41,322	MH732933	31	RNA-dependent RNA polymerase [Rhizoctonia solani mitovirus 12]

## Supplementary Materials

The following are available online at http://www.mdpi.com/1999-4915/10/12/707/s1, Figure S1: Stain of cross-section of the roots to confirm the AM fungal infection showing the (A) density of arbuscules as the small oval-shaped objects and (B) close-up view of hyphae and connected arbuscules.
