# Supplementary material for: Metatranscriptomic Analysis and In Silico Approach Identified Mycoviruses in the Arbuscular Mycorrhizal Fungus Rhizophagus spp
Source: Viruses. 2018 Dec 12;10(12):707. doi: 10.3390/v10120707 (PMC6316171; doi:10.3390/v10120707)
Supplement: Supplementary file 1 [file viruses-10-00707-s001.pdf]

Supplemental Material

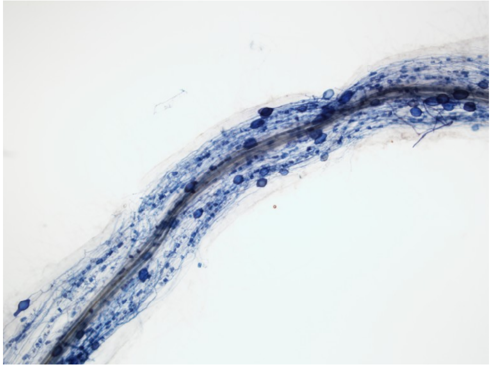

A

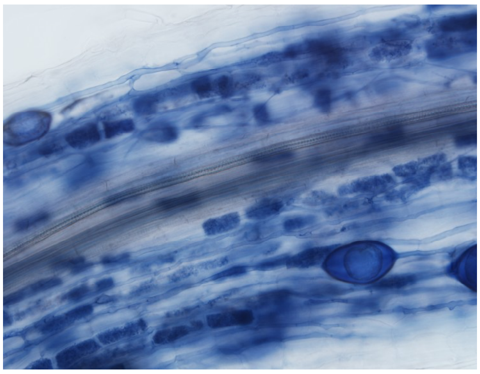

B

Figure S1 Stain of cross-section of the roots to confirm the AM fungal infection showing the (A) density of arbuscules as the small oval-shaped objects and (B) closeup view of hyphae and connected arbuscules.
